# Management and outcomes in pulmonary arterial hypertension patients with sepsis

**DOI:** 10.1186/s12890-024-03355-5

**Published:** 2024-10-28

**Authors:** Spencer Flynn, Haidee Chen, Russell Kerbel, Summer Gupta, Sonia Jasuja, Rajan Saggar, Richard Channick, Alexander Sherman

**Affiliations:** 1 David Geffen School of Medicine at UCLA, Los Angeles, CA USA; 2 Department of Medicine, University of California Los Angeles, Los Angeles, CA USA; 3 Division of Pulmonary and Critical Care Medicine, David Geffen School of Medicine at UCLA, 200 Medical Plaza, Suite 365, Los Angeles, CA 90095 USA

**Keywords:** Pulmonary arterial hypertension, Sepsis, Fluid resuscitation

## Abstract

**Background:**

Sepsis is a common cause of death in patients with pulmonary arterial hypertension (PAH). Treatment requires careful fluid management and hemodynamic support. This study compares patients with or without PAH presenting with sepsis with a focus on initial fluid resuscitation.

**Methods:**

This retrospective analysis compared adults with and without PAH admitted for sepsis at two academic hospitals between 2013 and 2022. Prior PAH diagnosis was verified by review of right heart catheterization data and sepsis present on admission was verified by chart review. Demographics, vital signs, laboratory values, imaging results, treatment approaches, and all-cause mortality data were obtained. Controls were propensity score weighted by age, sex, and Charlson Comorbidity index. Logistic regression models controlling for age and Charlson comorbidity indices were used to examine factors associated with survival.

**Results:**

Thirty patients admitted for sepsis with pre-existing PAH were compared to 96 matched controls. Controls received significantly more fluids at 24 h compared to PAH patients (median 0 mL v. 1216 mL, *p* < 0.001), while PAH patients were more likely to receive vasoactive medications (23.3% vs. 8.3%, *p* = 0.037). At 30 days, 7 PAH patients (23.3%) and 13 control patients (13.5%) had died (*p* = 0.376). PAH patients that received more fluids had decreased mortality (OR 0.31, 95% CI 0.11–0.92, *p* = 0.03) and patients who received fluids had shorter mean time to antibiotics (2.3 h v. 6.5 h, *p* = 0.04), although decreased time to antibiotics was not associated with mortality. Patients who received no fluids more often had previously identified right ventricular systolic dysfunction (62.5% v. 28.6%, *p* = 0.136).

**Conclusion:**

Patients with PAH and sepsis have high mortality and receive different treatments than controls, with more reliance on vasopressors and less on fluid resuscitation. PAH patients who received less fluids had higher mortality and those who received no fluids had a longer time to receiving antibiotics, indicating a potential delay in recognizing sepsis. Timely recognition of sepsis and dynamic decision-making around fluid resuscitation remains critical in this high-risk population.

**Supplementary Information:**

The online version contains supplementary material available at 10.1186/s12890-024-03355-5.

## Introduction

Pulmonary arterial hypertension (PAH) is a disease characterized by pulmonary arterial remodeling leading to increased pulmonary vascular resistance (PVR), right ventricular (RV) dysfunction, and right heart failure [1, 2]. Sepsis is the second most common cause of in-hospital death in PAH patients [3–5]. Recent data suggests that about 25% of patients with PAH who present for sepsis will die while in the hospital [3]. Prompt recognition and treatment of sepsis is vital in the management of this high-risk condition.

Sepsis causes numerous systemic changes with adverse effects on the cardiac and pulmonary systems. These changes include decreased right ventricular preload, increased right ventricular afterload, and reduced contractility, all in the setting of increased myocardial oxygen demand [6–14]. Each of these factors are further exacerbated in patients with PAH, with unique implications for management. Current management of sepsis in PAH patients is based on expert opinion rather than clinical data, and includes treating the underlying etiology, optimizing preload, reducing right ventricular afterload, and supporting myocardial function to achieve adequate systemic perfusion [15, 16]. Decisions around fluid management are particularly challenging in PAH patients with sepsis. Fluid resuscitation is recommended as part of initial resuscitation to restore preload in septic patients [17]. However, differentiating sepsis from other causes of acute on chronic right ventricular dysfunction is challenging given the often non-specific signs in early sepsis in patients with PAH. Furthermore, administering fluids to increase preload risks worsening right ventricular dilatation, ventricular interdependence, and coronary perfusion in the setting of sepsis-induced increased PVR [10, 15, 18]. This presents a major challenge in the management of sepsis in PAH patients as right ventricular volume overload can lead to impaired cardiac output, with catastrophic implications for the pulmonary and systemic circulations [15, 16]. 

Currently, there is little data describing clinical characteristics or treatment strategies for sepsis in this acutely ill population [17]. Current treatment recommendations for PAH patients presenting with sepsis are based on expert opinion rooted in clinical practice or the results of registry or administrative database studies with incompletely characterized cohorts, and so the recommendations are frequently vague or lack supporting data. This retrospective study investigates the treatment approaches and outcomes in a well-characterized cohort of patients with PAH verified by right heart catheterization who presented with sepsis between 2013 and 2022, with a particular focus on initial fluid resuscitation. We hypothesized that PAH patients would receive less fluids and that increased fluid resuscitation would be associated with worse outcomes.

## Methods

We conducted a retrospective cohort study of patients with PAH, compared to controls without PAH, who all presented for sepsis between 2013 and 2022 at two academic hospitals. PAH patients and controls were adults identified by an automated database search. Sepsis present on admission was identified by ICD-10 diagnosis codes. PAH patients were initially identified by ICD-10 codes for pulmonary arterial hypertension preceding their hospital admission. The specific ICD-10 codes used are listed in the Supplemental. PAH patients were admitted between October 1, 2015 – February 1, 2021, and controls were admitted between January 1st, 2013 and January 1st, 2022. Controls were enrolled in a 3:1 fashion compared to cases (PAH patients admitted with sepsis).

After PAH patients and controls were identified, all subsequent data was obtained via manual chart review. Sepsis was defined as the presence of at least two of four systemic inflammatory response syndrome (SIRS) criteria within 24 h of admission and a suspected source of infection. Sequential Organ Failure Assessment (SOFA) scores were not used as the electronic health records system used identifies sepsis through SIRS criteria and it was not feasible to retrospectively calculate accurate SOFA scores for many patients. PAH patients were excluded for the following: lung and/or heart transplant, current left ventricular assist device, or missing/incomplete right heart catheterization data. Both PAH patients and controls were required to have at least 6 months of follow up data or to have died within 6 months of admission. Additional data obtained through chart review included demographics, vital signs, laboratory values, medications used prior to and during admission, intravenous fluids administered during the first 24 and 48 h of admission, echocardiogram reports, and right heart catheterization reports. Patients were categorized by right heart catheterization data as pre-capillary or mixed pre-post capillary pulmonary hypertension (PH) using criteria from the 6th World Health Symposium on Pulmonary Hypertension [21]. Acute kidney injury was defined using Kidney Disease Improving Global Outcomes (KDIGO) criteria [22]. Additionally, patients were identified as being in septic shock using Sepsis-3 criteria of requiring vasopressor support to maintain a MAP > 65, and a lactate > 18 mg/dL, and patients were also identified who met the Septic Shock Management Bundle (SEP-1) bundle criteria for fluid resuscitation within 3 h of admission by presenting with initial SBP < 90, MAP < 65, or lactate > 18 mg/dL [19, 20].

The study was approved by our institutional review board, Office of the Human Research Protection Program (OHRPP) IRB#22–000226. Clinical trial number is not applicable.

### Outcomes and subgroups

Primary outcomes included mortality during or within 30 days of admission. Secondary outcomes included hospital length of stay, intensive care unit (ICU) length of stay, and mechanical ventilator use during admission. Patients were stratified for subgroup analyses by mortality during or within 30 days of admission, receipt of fluid resuscitation within 24 h of admission, and by RV systolic function on pre-admission echocardiogram.

### Statistical analysis

Propensity score weighting by age, sex, and Charlson comorbidity index was performed for comparisons between patients and controls. The propensity score distribution was inspected visually via a histogram and did not include extreme values that could skew the analysis (see Supplemental Fig. 1). Differences between the groups were evaluated by inverse probability weighted regression adjustments. Septic shock was not included in the propensity scoring since a primary question in this study was differences in vasopressor versus fluid use, and it was felt that the septic shock variable would be highly collinear with vasopressor use. Clinical characteristics and treatment approaches for PAH patients stratified by mortality, fluid resuscitation, and baseline RV systolic function were summarized. Differences between the PAH subgroups were measured by Kruskal-Wallis and Chi-square tests, as appropriate to the variable distribution. Several logistic regression models were generated to determine the relationships between mortality and various predictors, including fluids given, vasopressor use, time to antibiotics, number of prior PAH therapies, mechanical ventilation, kidney injury, and various proxies of right ventricular dysfunction. All models were adjusted for age, sex, and Charlson comorbidity indices. *P*-values < 0.05 were considered statistically significant. All statistical analyses were performed with R version 4.0.5. (R Foundation for Statistical Computing, Vienna, Austria).

## Results

**Fig. 1:**
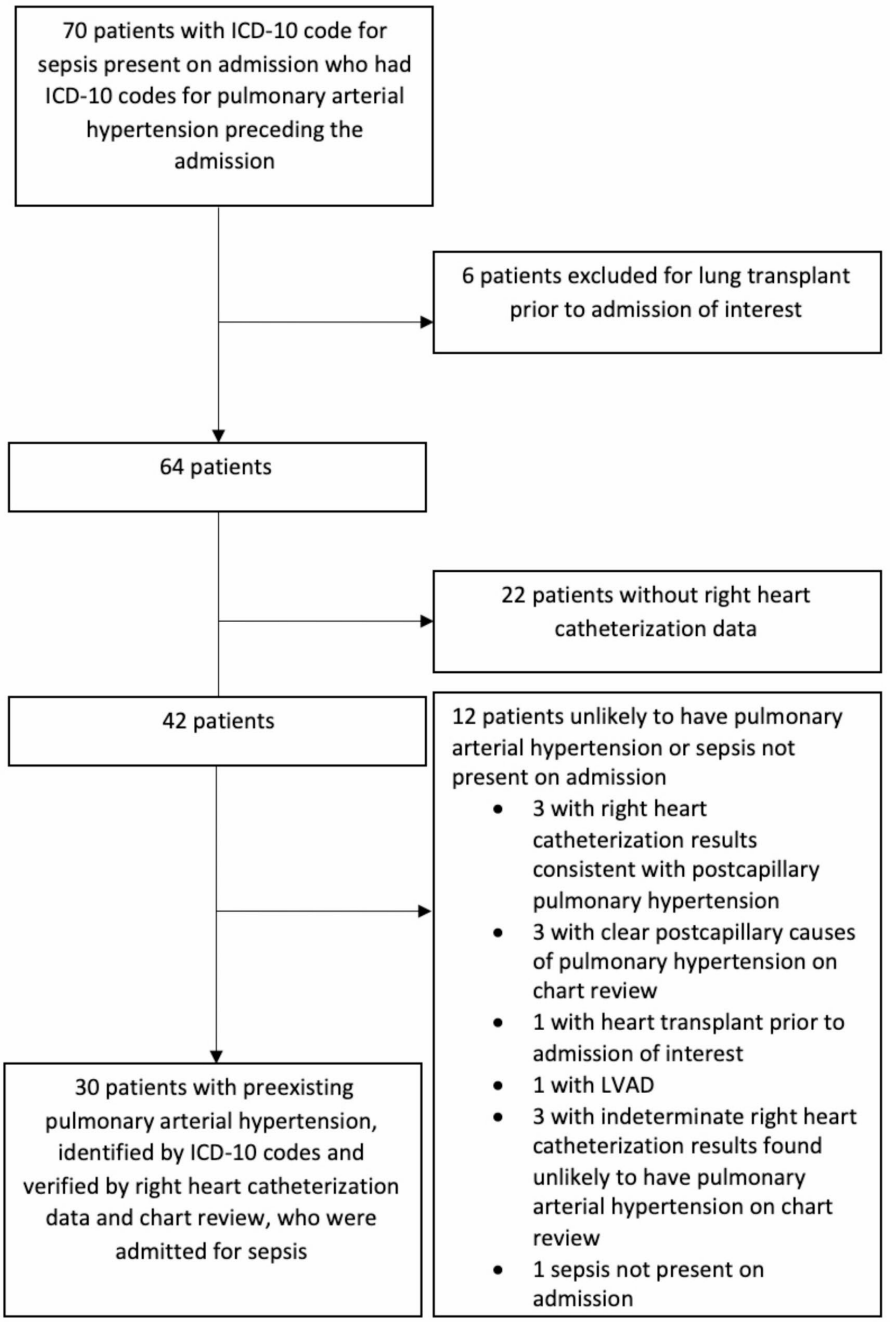
Flow diagram of inclusion and exclusion of subjects

There were 70 patients identified by ICD-10 codes as having PAH and sepsis present on admission. Thirty cases were included after verification of PAH and sepsis present on admission (Fig. 1). There were also 96 age and sex-matched controls reviewed. Among PAH patients, the average age was 57.2 years, 80% were female, 50% were white, 80% were on PAH-specific therapy, and 30% were in septic shock (Table 1). Non-propensity weighted data is reported in Supplemental Tables 1 and the etiology of sepsis is reported in Supplemental Table 2. Pneumonia and gastrointestinal infections were the most common infections in both groups, and PAH patients were significantly more likely to have endocarditis (6.7% v. 0%, *p* = 0.011) or central line infections (16.7% v. 0%, *p* < 0.001) as causes of infection compared to controls.

**Table 1:** Propensity score weighted demographics and clinical characteristics of patients with pulmonary arterial hypertension admitted for sepsis compared to controls

	PAH patients (*n* = 30)	Controls (*n* = 96)	*P*-value
Age	57.2 ± 16.5	57.6 ± 16.3	0.361
Sex, Female	24 (80%)	73 (76%)	0.522
Race			
White Black Asian Other	15 (50%) 6 (20.0%) 1 (3.3%) 8 (26.6%)	58 (60.4%) 5 (5.0%) 11 (12.1%) 22 (22.6%)	0.399 **0.031** **0.008** 0.818
Charlson comorbidity index	4.0 ± 2.6	3.9 ± 2.5	0.237
Presenting vitals and laboratory data			
MAP, mmHg Respiratory rate, per minute Temperature, Fahrenheit Heart rate, per minute SpO2, % WBC, x10^3^/μL Lactate, mg/dL Creatinine, mg/dL BNP, pg/mL	76.2 ± 16.8 21.5 ± 5.2 98.8 ± 1.7 94.7 ± 21.6 91.9 ± 7.1 9.7 ± 5.3 16.7 ± 12.1 1.97 ± 2.08 876.4 ± 727.9	90.6 ± 16.1 20.1 ± 5.7 99.1 ± 2.2 100.8 ± 21.2 95.4 ± 7.2 14.2 ± 10.3 20.1 ± 18.8 1.47 ± 1.39 600.0 ± 768.2	**< 0.001** 0.133 0.391 0.157 **0.014** **0.002** 0.191 0.127 0.139
Blood culture, positive at 24 h	9 (30%)	24 (25.2%)	0.760
Septic shock	9 (30%)	8 (8.0%)	**0.006**
SEP-1 Bundle	14 (46.7%)	34 (35.5%)	0.224
Time to antibiotics, hours	7.4 ± 5.0	5.0 ± 11.3	0.370
PH medication during admission, categorical	26 (86.7%)	N/A	N/A
Received parenteral PH medication during admission	10 (33.3%)	N/A	N/A
Baseline PH medications used, categorical	24 (80%)	N/A	N/A
Number of baseline PH medications used			
0 1 2 3	6 (20%) 6 (20%) 5 (16.7%) 13 (43.4%)	N/A	N/A
Maximal supplemental oxygen use at 24 h			
ETT PPV HFNC NRB NC Room air	0 1 (3.3%) 10 (33.6%) 2 (6.6%) 13 (43.3%) 4 (13.3%)	6 (6.3%) 4 (4.2%) 2 (2.1%) 8 (8.3%) 31 (32.3%) 45 (46.9%)	**0.012** 0.440 **< 0.001** 0.594 0.276 **< 0.001**

Abbreviations: MAP = mean arterial pressure, SpO2 = oxygen saturation, WBC = White blood cell count, BNP = brain natriuretic peptide, PH = pulmonary hypertension, ETT = endotracheal intubation, PPV = positive pressure ventilation, HFNC = high flow nasal cannula, NRB = non-rebreather mask, NC = nasal cannula

Cases and controls were propensity score weighted by Charlson Comorbidity index, age, and sex

All numeric values are reported as mean ± SD. *P*-values are from Inverse Probability Weighted Regression Adjustment

All vital signs and lab values reported are the earliest available values during the qualifying encounter for sepsis. *p < 0.05, **p < 0.01, ***p < 0.001

### Hemodynamic measurements of cases with PAH and sepsis

Among PAH patients, the average most recent right heart catheterization was 15 months prior to admission, and the most recent echocardiogram was 7 months prior to admission (Table 2). There were 20 patients with pre-capillary pulmonary hypertension, 6 with mixed pre-post capillary hypertension, and 4 categorized as neither but who on chart review had PAH. The mean ± SD pulmonary artery pressure was 42.1 ± 12.0 mmHg, with a pulmonary capillary wedge pressure of 11.9 ± 5.8 mmHg and a pulmonary vascular resistance of 7.0 ± 4.2 Wood Units (WU). The mean cardiac index was 2.9 ± 1.1 L/min/m^2^. The mean left ventricular ejection fraction was 72.1 ± 5.7%, with the majority (66.7%) having normal LV systolic function. There were 13 patients (43.3%) with flattened interventricular septa and 12 patients (40%) with dilated inferior vena cava. There were 14 (46.7%) patients with at least mild RV systolic dysfunction on prior echocardiogram (subgroup analysis of treatment approaches and mortality in this subgroup is included in Supplemental Table 3).

**Table 2:** Hemodynamic measurements prior to admission of patients with pulmonary arterial hypertension

	All PAH patients (*n* = 30)	Precapillary PH group (*n* = 20)	Mixed PH group (*n* = 6)	Uncategorized group (*n* = 4)
Sex, female	24 (80%)	17 (85%)	4 (67%)	3 (75%)
Time from RHC to admission for sepsis, months	15.2 ± 16.9	11.8 ± 8.2	13.8 ± 9.8	34.4 ± 40.1
Mean RAP, mmHg	8.9 ± 5.3	7.8 ± 4.1	14.8 ± 6.6	5.5 ± 1.3
Mean PAP, mmhG	42.1 ± 12.0	41.3 ± 11.8	50.6 ± 10.9	33.3 ± 7.9
PCWP, mmHg	11.9 ± 5.8	9.3 ± 4.1	21.4 ± 2.5	13 ± 1.4
CO, thermodilution, L/min	5.1 ± 2.1	4.4 ± 1.3	4.8 ± 1.4	9.0 ± 1.6
CO, Fick, L/min	4.6 ± 1.0	4.5 ± 1.1	4.7 ± 0.3	NA
CI, thermodilution, L/min/m^2^	2.9 ± 1.1	2.7 ± 0.8	2.4 ± 0.7	5.2 ± 0.7
CI, Fick, L/min/m^2^	2.3 ± 0.4	2.4 ± 0.4	2.2 ± 0.1	NA
PVR, WU	7.0 ± 4.2	8.2 ± 4.2	6.0 ± 3.2	2.2 ± 0.6
Time from TTE data to admission for sepsis, months	6.9 ± 11.3	4.1 ± 4.0	7.5 ± 6.7	20.4 ± 27.8
LA size				
Normal Enlarged	16 (53.3%) 14 (46.7%)	11 (55%) 9 (45%)	2 (33.3%) 4 (66.6%)	3 (75%) 1 (25%)
LVEF, %	72.1 ± 5.7	70 ± 8.7	73.3 ± 2.9	75 (NA)
LV systolic function				
Normal Mildly reduced Hyperdynamic	20 (66.7%) 1 (3.3%) 9 (30%)	15 (75%) 1 (5%) 4 (20%)	3 (50%) 0 3 (50%)	2 (50%) 0 2 (50%)
IV septum description, flattened	13 (43.3%)	12 (60%)	1 (16.7%)	0
IV septum diameter, cm	1.1 ± 0.3	1.1 ± 0.4	1.1 ± 0.3	1.2 ± 0.3
Diastolic function description				
Normal Grade I dysfunction Grade II dysfunction Not described	12 (40%) 9 (30%) 1 (3.3%) 8 (26.7%)	9 (45%) 5 (25%) 0 6 (30%)	1 (16.7%) 3 (50%) 0 2 (33.3%)	2 (50%) 1 (25%) 1 (25%) 0
RV size				
Normal Mildly enlarged Moderately enlarged Severely enlarged	10(33.3%) 8 (26.7%) 3 (10%) 9 (30%)	4 (20%) 7 (35%) 2 (10%) 7 (35%)	4 (66.7%) 0 0 2 (33.3%)	2 (50%) 1 (25%) 1 (25%) 0
RV systolic function				
Normal Mildly reduced Moderately reduced Severely reduced Not commented	15 (50%) 5 (16.7%) 3 (10%) 6 (20%) 1 (3.3%)	8 (40%) 3 (15%) 3 (15%) 5 (25%) 1 (5%)	5 (83.3%) 0 0 1 (16.7%) 0	2 (50%) 2 (50%) 0 0 0
TAPSE, cm	1.9 ± 0.5	1.9 ± 0.6	1.7 ± 0.4	2.3 ± 0.3
DTI, cm/s	12.7 ± 2.6	12.0 ± 1.8	12.3 ± 2.4	17.8 ± 1.1
Tricuspid regurgitation description				
Trace Mild Mild-moderate Moderate Moderate-severe Severe Not commented	8 (26.7%) 6 (20%) 5 (16.7%) 3 (10%) 4 (13.3%) 3 (10%) 1 (3.3%)	5 (25%) 4 (20%) 4 (20%) 2 (10%) 2 (10%) 3 (15%) 0	1 (16.7%) 0 1 (16.7%) 1 (16.7%) 2 (33.3%) 0 1 (16.7%)	2 (50%) 2 (50%) 0 0 0 0 0
Tricuspid regurgitation velocity, m/s	3.6 ± 0.9	3.6 ± 1.0	3.8 ± 0.7	3.0 ± 0.6
Estimated RVSP/PASP	63.4 ± 28.4	66.1 ± 29.4	71 ± 23.8	36.7 ± 14.7
IVC dilated, yes	12 (40%)	9 (45%)	2 (33.3%)	1 (25%)
IVC respiratory change, < 50%	19 (63.3%)	12 (60%)	6 (100%)	1 (25%)
Pericardial effusion				
None or trace Small Moderate Large	25 (83.3%) 2 (6.7%) 2 (6.7%) 1 (3.3%)	17 (85%) 1 (5%) 1 (5%) 1 (5%)	4 (66.7%) 1 (13.3%) 1 (13.3%) 0	4 (100%) 0 0 0

Abbreviations: RHC = right heart catheterization, RAP = right atrial pressure, PAP = pulmonary artery pressure, PCWP = pulmonary capillary wedge pressure, CO = cardiac output, CI = cardiac index, PVR = pulmonary vascular resistance, WU = Wood units, LA = left atrium, LVEF = left ventricular ejection fraction, IV = interventricular, RV = right ventricle, TAPSE = tricuspid annular plane systolic excursion, DTI = doppler tissue echocardiography, RVSP/PASP = right ventricular systolic pressure/pulmonary artery systolic pressure, IVC = inferior vena cava.

All numeric variables are mean ± SD unless otherwise indicated.

Hemodynamic categories were defined by World Health Symposium on Pulmonary Hypertension criteria: precapillary is mPAP > 20mmHG, PCWP ≤ 15mmHG, and PVR ≥ 3WU. Mixed is mPAP > 20mmHG, PCWP > 15, PVR ≥ 3WU. Uncategorized has hemodynamic values that do not fall into any group

### Treatment approaches and mortality

**Fig. 2:**
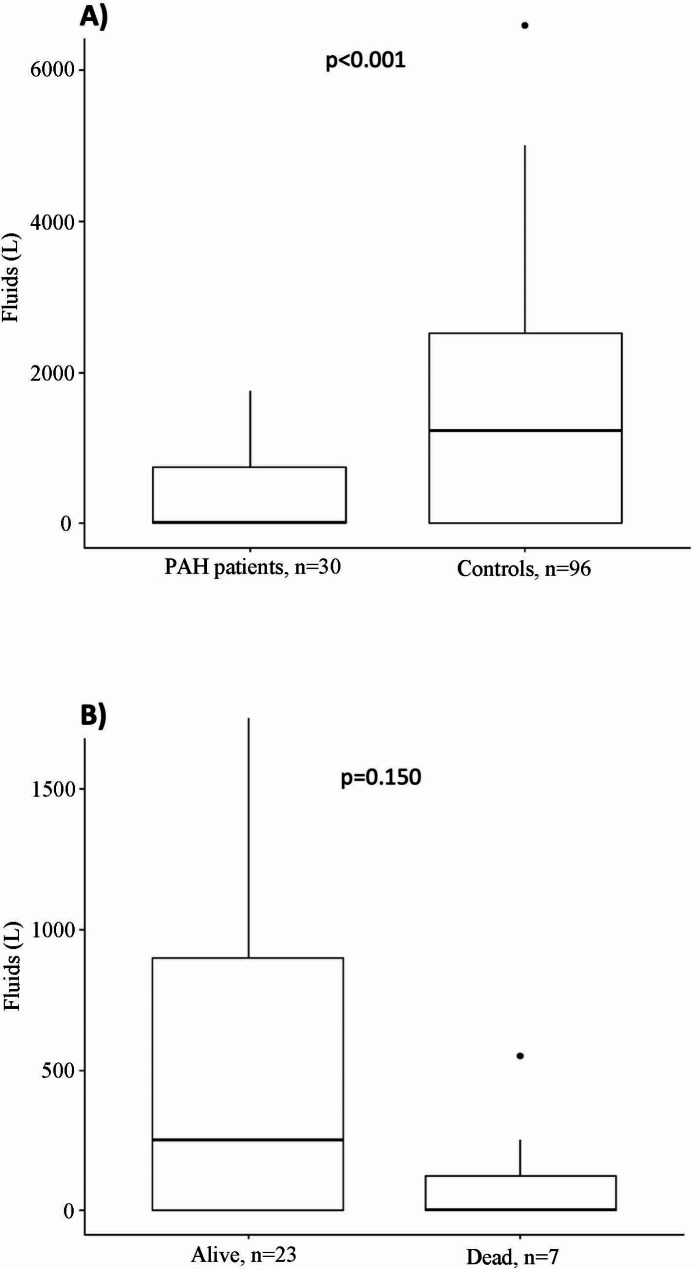
Fluid boluses given. (**A**) Fluid boluses given in 24 h to PAH patients compared to controls. (**B**) Fluid boluses given in 24 h to PAH patients who survived for 30 days compared to PAH patients who died within 30 days

PAH patients received lower volume intravenous fluids (median 0, IQR 0-750 mL v. 1216, IQR 0-2560 mL at 24 h, *p* < 0.001) (Fig. 2), and were more likely to receive vasopressors (23.3% v. 8.3% at 24 h, *p* = 0.037, Table 3). They also had non-significantly increased hospital lengths of stay (mean ± SD, 31.6 ± 48.5 days v. 17.5 ± 30.7 days, *p* = 0.127), mechanical ventilation (30% v. 22.9%, *p* = 0.433), and mortality within 30 days (23.3% v. 13.5%, *p* = 0.376). Three month and 6-month mortality were also non-significantly higher in PAH patients (23.3% v. 14.5%, *p* = 0.400, and 26.7% v. 16.7%, *p* = 0.342, respectively). See Supplemental Table 4 for non-propensity weighted data, which had similar findings.

**Table 3:** Treatment approaches and outcomes in pulmonary arterial hypertension patients admitted for Sepsis compared to controls

	All PAH patients (*n* = 30)	Controls(*n* = 96)	*P*-value
IV fluid boluses in first 24 h, mL, median (IQR)	0 (0-750)	1216 (0-2560)	**< 0.001**
IV fluid boluses in first 48 h, mL, Median (IQR)	250 (0-970)	1374 (133–3000)	**< 0.001**
Vasopressor use in first 24 h	7 (23.3%)	8 (8.3%)	**0.037**
Vasopressor use in first 48 h	10 (33.3%)	8 (8.3%)	**0.004**
Hospital length of stay, days	31.6 ± 48.5	17.5 ± 30.7	0.127
Mechanical ventilation during admission	9 (30%)	22 (22.9%)	0.433
Mortality during admission or within 30 days	7 (23.3%)	13 (13.5%)	0.376

Abbreviations: IV = intravenous, PH = pulmonary hypertension

PAH patients and controls were propensity score weighted by Charlson Comorbidity index, age, and sex

All numeric variables are mean ± SD unless otherwise indicated. *P*-values are from Inverse Probability Weighted Regression Adjustment

### Mortality associations

PAH patients who died during or within 30 days of admission were older (median, IQR 74 years (67-77.5) vs. 55 years (48.5–59), *p* = 0.017) and had higher Charlson comorbidity indices (6 (5.5–7.5) vs. 4 (2-5.5), *p* = 0.019) compared to those who survived (Table 4). PAH patients who died were more likely to require high flow nasal cannula within 24 h of admission (57.1% v. 21.7%, *p* = 0.030). They also had significantly lower heart rates and temperature on admission compared to those who survived. Overall, those who died had similar rates of septic shock, similar RV systolic function, and similar baseline hemodynamics. They also had similar rates of PAH therapy prior to and during admission, vasopressor use, mechanical ventilation, hospital length of stay, and likelihood of being in the ICU. They had non-statistically significant increased likelihood of AKI present on admission (57.1% v. 26.1%), time to antibiotics (4.5 h IQR 2.5–7.6 v 2.7 h IQR 1.3–5.1) (Table 4), and received less intravenous fluid in the first 24 h (0 mL IQR 0-125 v. 250 mL IQR 0-900) (Fig. 2).

**Table 4:** Characteristics and treatment approaches of patients with pulmonary arterial hypertension admitted for sepsis by mortality

	Alive (*n* = 23)	Deceased (*n* = 7)	*P*-value
Age	55 (48.5–59)	74 (67-77.5)	**0.017**
Sex, female	19 (82.6%)	5 (71.4%)	0.914
Race			
White Black Asian Other	White 9 (39.1%) Black 6 (26.1%) Other 7 (30.4%)	White 6 (85.7%) Black 1 (14.3%)	0.166
Charlson comorbidity index	4 (2-5.5)	6 (5.5–7.5)	**0.019**
Presenting vitals and laboratory data			
MAP, mmHg Respiratory rate, per minute Temperature, Fahrenheit Heart rate, per minute SpO2, % WBC, x10^3^/μL Serum lactate, mg/dL BNP, pg/mL	74.7 (66.8–88.3) 20 (18–25) 98.5 (98-100.5) 103 (88.5-110.5) 95 (92.5–96) 7.1 (6-11.3) 13 (8-19.5) 494 (223.2-1265.8)	68 (62.8–79.7) 18 (17–19) 97.5 (97.3–97.9) 74 (68-85.5) 90 (87-94.5) 11.1 (10.1–12.7) 14 (10-16.5) 890 (356.5–1378.0)	0.249 0.156 **0.006** **0.013** 0.209 0.249 0.702 0.619
Blood culture, positive	7 (30.4%)	2 (28.6%)	0.349
Septic shock	7 (30.4%)	2 (28.6%)	0.928
Respiratory infectious source	8 (34.8%)	5 (71.4%)	0.201
SEP-1 Bundle criteria	11 (47.8%)	3 (42.9%)	1.000
PH medication during admission	19 (82.6%)	7 (100%)	0.582
Received parenteral PH medication during admission	14 (60.9%)	5 (71.4%)	0.952
Baseline PH medication use, yes	18 (78.3%)	7 (100%)	0.440
Number of baseline PH medications used			
0 1 2 3	0 (21.7%) 1 (17.4%) 2 (17.4%) 3 (43.5%)	0 3 (42.9%) 2 (28.6%) 2 (28.6%)	0.302
Supplemental oxygen use at 24 h			
None NC NRB HFNC	4 (17.4%) 13 (56.5%) 1 (4.3%) 5 (21.7%)	0 (0%) 2 (14.3%) 1 (14.3%) 4 (57.1%)	**0.030**
Time to antibiotics, hours	2.7 (1.3–5.1)	4.5 (2.5–7.6)	0.441
PCWP, mmHg	11 (8–14)	12 (8.5–14.5)	0.459
Mean RAP, mmHg	7 (5-12.5)	10 (8.5–10.5)	0.403
Mean PAP, mmHg	41 (32-49.8)	45 (40-48.5)	0.556
PVR, WU	7.8 (3.3–9.7)	5.8 (4.2–6.8)	0.351
CI, thermodilution, L/min/m^2^	2.6 (2.1–3.4)	2.9 (2.9-3.0)	0.301
RV systolic function reduced	11 (47.8%)	3 (42.9%)	1.000
Change in lactate	-5.5 (-9.2, -0.8)	-7 (-26, -7)	0.148
Change in creatinine, 24 h	-0.1 (-0.2-0.1)	-0.1 (-0.9-0.1)	0.683
AKI present on admission	6 (26.1%)	4 (57.1%)	0.146
AKI resolved by 48 h	6 (27.3%)	2 (28.6%)	0.395
Mechanical ventilation used during admission	7 (30.4%)	2 (28.6%)	1.000
IV fluid boluses in first 24 h, mL	250 (0-900)	0 (0-125)	0.150
IV fluid boluses in first 48 h, mL	250 (0-1500)	0 (0-400)	0.183
Diuretic use in first 24 h	10 (43.5%)	4 (57.1%)	0.840
Diuretic use in first 48 h	14 (60.9%)	4 (57.1%)	1.000
Vasopressor use in first 24 h	5 (21.7%)	2 (28.6%)	1.000
Vasopressor use in first 48 h	8 (34.8%)	2 (28.6%)	1.000
Hospital length of stay	15 (8-38.2)	11 (7.5–20.5)	0.628
ICU stay during admission	14 (60.9%)	4 (57.1%)	1.000
ICU length of stay	23.5 (13.5–30)	21 (11-59.1)	1.000

Abbreviations: MAP = mean arterial pressure, SpO2 = oxygen saturation, WBC = White blood cell count, RAP = right atrial pressure, PAP = pulmonary artery pressure, PCWP = pulmonary capillary wedge pressure, CI = cardiac index, PVR = pulmonary vascular resistance, WU = Wood units, ICU = intensive care unit

All vital signs and lab values reported are the earliest available values during the qualifying encounter for sepsis

All numeric values are reported as median (IQR) and *p*-values are from Kruskal-Wallis tests. *p < 0.05, **p < 0.01, ***p < 0.001

### Logistic regression models of survival among patients with pulmonary arterial hypertension hospitalized for sepsis

In the logistic regression models, increased fluid resuscitation at 24–48 h were both significantly associated with decreased mortality (Table 5, OR and 95% CI for each 250 mL of fluid given 0.31 (0.11–0.91), *p* = 0.030, and 0.29 (0.10–0.85), *p* = 0.024, respectively). A number of variables were non-significantly associated with higher likelihood of mortality, including vasopressor use, higher number of PH medications used at baseline, reduced RV systolic function, acute kidney injury on presentation, ICU stay during admission, and mechanical ventilation during admission. Time to antibiotics was not associated with survival.

**Table 5:** Logistic regression models of Survival among patients with pulmonary arterial hypertension hospitalized for sepsis

	OR (95% CI)
Quantity fluid boluses given at 24 h, per 250 cc	**0.31 (0.11–0.92)**,**p = 0.03**
Quantity fluid boluses given at 48 h, per 250 cc	**0.29 (0.10–0.85)**,**p = 0.02**
Vasopressor use at 24 h	55.3 (1.5-10989), *p* = 0.06
Vasopressor use at 48 h	4.47 (0.32–116), *p* = 0.29
PH medication use at baseline, ordinal of 0, 1, 2, or > = 3 medications	3.84 (0.85–48.26), *p* = 0.16
Time to antibiotics, hours	1.02 (0.83–1.18), *p* = 0.75
Mechanical ventilation during admission	2.25 (0.17–31.71), *p* = 0.51
TAPSE, continuous	4.42 (0.12–703), *p* = 0.47
RV systolic function at least mildly impaired	1.80 (0.16-25.0), *p* = 0.63
AKI on presentation	3.63 (0.34–51.7), *p* = 0.29
ICU stay during admission	3.24 (0.38–48.1), *p* = 0.32

Abbreviations: TAPSE = tricuspid annular plane systolic excursion, RV = right ventricle

All values reported are odds ratios generated from logistic regression analyses adjusted for age, sex, and Charlson Comorbidity Index at admission. Each of the variables listed in this table were run in a separate model, for 11 total logistic regression models

Additional logistic regression models were generated to identify predictors of receiving intravenous fluid resuscitation (see Supplemental Table 5). A significant predictor for increased likelihood of receiving fluids was higher initial lactate (OR and 95% CI 1.23 (1.04–1.55,) *p* = 0.049). Impaired RV systolic function on previous echocardiogram also predicted lower likelihood of receiving fluids (OR and 95% CI 0.14, (0.02–0.99), *p* = 0.050).

### Fluid resuscitation associations

There were 14 PAH patients who received fluid resuscitation within the first 24 h, compared to 16 patients who received no fluids (Supplemental Table 6). Both groups had similar presenting vitals, laboratory findings, and sepsis severity. Only 28.6% of those who received fluids were on three PH drugs compared to 50% of patients who did not receive fluids, and those who received fluids were less likely to be on parental PH medication (35.7% v. 87.5%, *p* = 0.011), had lower mean pulmonary artery pressures (37 mmHg IQR 27–45 vs. 45.5 mmHg IQR 40–52, *p* = 0.037) and were less likely to have reduced RV systolic function (28.6% v. 62.5%, *p* = 0.136). Those who received fluids had shorter time to antibiotics (2.3 IQR 1.2–4.5 h vs. 6.5 IQR 2.5–19.1 h, *p* = 0.041) and were less likely to receive diuretics in the first 48 h of their hospital stay (35.7% v. 81.2%, *p* = 0.030). Those who received fluids also trended towards resolution of AKI within 48 h (100% v. 50%) and greater improvement in lactate (median, IQR 6.5 mg/dL (0.8–10.2) v. 5 (4–6)). There was 1 patient who received fluids that died (7.1%), compared to 6 of the patients who did not receive fluids (37.5%), although this difference was not significant (*p* = 0.126).

## Discussion

This retrospective study compared patients with PAH presenting with sepsis to controls without PAH to characterize clinical presentation and treatment approach with a focus on fluid resuscitation. The key findings include (1) PAH patients with sepsis had a high mortality rate of 23.3%, (2) PAH patients received less intravenous fluid resuscitation and were more likely to receive vasopressors (3) variables associated with increased mortality included age, Charlson comorbidity index, low heart rate, low temperature, and need for high flow nasal cannula within 24 h of admission, while increased volume of fluid resuscitation in the first 24 h of admission was associated with lower mortality.

PAH patients had a high mortality rate of 23.3% compared to 13.5% among controls (*p* = 0.376). The lack of statistical difference may be due in part to lack of power, but is similar to a large database study demonstrating a mortality rate of 25% in patients with PAH and sepsis [1]. He et al. examined 285 patients with sepsis and PAH and found lower mortality of 16.3%, however these patients may have been less ill, suggested by a lower rate of vasopressor use in both PAH patients and controls (3.1% and 2.3% use, compared to 33.3% and 8.3%, respectively, in this study) [14]. Notably, the absolute difference in mortality between PAH patients and controls in our study was about 10% even with propensity score weighting of controls by Charlson comorbidity index. This suggests that PAH may increase mortality risk in sepsis beyond the comorbidity points assigned to heart failure in the index. Further, it is worth noting twice as many PAH patients who died had respiratory sources of infection compared to those who survived (71% v. 35%, see Table 4) although this did not reach statistical significance. This suggests that pneumonia as the etiology of sepsis may be especially morbid in patients with PAH, and makes physiologic sense as pronounced hypoxemia, hypercapnia, and increased pulmonary resistance pressures in pulmonary disease are known to worsen PAH [15]. 

In this study, controls received larger IV fluid volumes within 24 h, while PAH patients were significantly more likely to receive vasopressors within 24 h. This suggests a difference in the approach to initial resuscitation in patients with PAH and sepsis. The 2021 Surviving Sepsis Guidelines recommend fluid resuscitation as first line in patients with sepsis and organ hypoperfusion or septic shock, yet PAH patients with hypotension in this study frequently received pressors as the first treatment rather than IV fluid [17]. For example, 5 out of 9 of the PAH patients who were categorized as in septic shock in fact did not receive any fluids.

This difference in management may in part be due to the challenge facing physicians in differentiating sepsis from non-infectious cardiac decompensation in patients with PAH. While all patients presented with at least two of four SIRS criteria, only about 40% of this cohort had positive blood cultures supporting a definitive infectious etiology. These findings highlight the diagnostic challenge that underlying PAH represents in the setting of organ malperfusion or hypotension, and may complicate the optimal volume management strategy given the risk of RV dysfunction with IV fluid administration [15, 23].

We found that fluid resuscitation may be beneficial in select PAH patients, as increased fluid boluses were associated with lower mortality in our cohort. It is worth noting that two recent randomized controlled trials of patients with severe sepsis or septic shock found no difference in mortality between restrictive or liberal fluid resuscitation strategies [24, 25]. However, both studies only included patients who were very ill with either severe sepsis or septic shock, and considerably more fluids were administered in the restrictive arm (mean 1.27 L and over 2 L, respectively) than were administered to the PAH patients in the present study (mean 0.41 L), and so their results likely do not generalize to our cohort. In the present study, IV fluids were associated with decreased mortality, non-significantly higher likelihood of resolution of AKI and improvement in lactate levels (Supplemental Table 4). Notably, these PAH patients had similar presenting vital signs, brain natriuretic peptide levels, and SEP-1 bundle criteria rates compared to controls. Fluid resuscitation was also associated with significantly early administration of antibiotics, suggesting earlier recognition of sepsis in patients who received fluids and which may underly some of the improvement in mortality, although interestingly time to antibiotics was not associated with improved mortality in our cohort [26, 27]. PAH patients who did not receive fluids had lower lactate levels, higher mean pulmonary artery pressures and trended towards greater RV dysfunction on transthoracic echocardiogram, which may reflect individualized decisions around the fluid management strategy and mortality given the known risks of fluid overload in this population.

Taken together, these findings suggest that systematic fluid restriction may be harmful, but also highlight the importance of careful hemodynamic evaluation and individualized risk-benefit assessment. As there are no current official guidelines for sepsis management in patients with pulmonary arterial hypertension, these findings provide information of clinical relevance and suggest that PAH patients with sepsis can be initially treated similarly to the general population of patients discussed in Surviving Sepsis guidelines, This includes initial treatment with early antibiotics and fluid administration, with vasopressors reserved for patients who show signs that they would not tolerate fluid administration (e.g. on bedside ultrasonography or physical assessment of fluid status) [17]. We also found that relatively small amounts of fluid may be of significant benefit in PAH patients with sepsis, unlike general sepsis patients who are recommended to receive 30 cc/kg of crystalloids in sepsis. Ultimately, our sample size was too small to arrive at concrete recommendations or to explore dose-response relationships, and fluid management decisions in this high risk population should be guided by individualized hemodynamic evaluations.

Limitations to this study include its retrospective nature, small sample size, and inability to obtain accurate SOFA scores, necessitating use of SIRS criteria to identify sepsis. Presenting volume status and any beside evaluation of cardiac function were not available. Propensity score weighting was used to help reduce confounding between PAH patients and controls, but this weighting could only use two variables (age and Charlson comorbidity index) due to statistical power considerations, and residual confounders are likely. Multiple confounders could also underly the finding of increased fluid resuscitation and improved mortality, particularly that patients with worse RV dysfunction may be both less likely to receive IV fluids given concerns of right ventricular dilatation and may also have higher mortality due to their cardiac dysfunction. This is a bicentric study of two academic centers, which may not be representative of broader clinical settings and so the possibility of a center effect cannot be excluded.

To our knowledge, this is the first study of PAH and sepsis that incorporated right heart catheterization data and chart review to generate a validated, well-characterized cohort of patients with PAH and sepsis. The few published studies on PAH and sepsis used large scale administrative databases or registries and lacked objective confirmation of PAH and sepsis [13, 14]. It is notable that our initial database search using ICD-10 codes generated 70 patients identified as having PAH and sepsis on presentation, but that after chart review only 30 patients were found to meet inclusion criteria. Further, while our small sample size was a limitation in terms of statistical power, it also allowed for complete phenotyping of each case.

## Conclusion

This was a retrospective study characterizing patients with PAH who presented with sepsis compared to propensity weighted controls without PAH. Patients with underlying PAH had high mortality, received lower volume of fluids during initial resuscitation, and had higher rates of vasopressor use compared to controls. Among PAH patients, those who received fluid resuscitation had improved mortality and shorter time to antibiotics. These findings reflect the challenges around early fluid resuscitation in PAH patients presenting with sepsis, weighing the risks of precipitating RV decompensation against under-resuscitation. Individualized assessment around fluid administration and dynamic hemodynamic evaluation is warranted. These findings also suggest that early identification of sepsis in these patients is critical in shortening time to antibiotics. Further research is needed to help guide decision-making around early fluid management in patients with PAH presenting with sepsis.

## Electronic supplementary material

Below is the link to the electronic supplementary material.

Supplementary Material 1

## Data Availability

The data that support the findings of this study are not openly available due to reasons of sensitivity and are available from the corresponding author upon reasonable request.

## Abbreviations

AKI: Acute kidney injury
KDIGO: Kidney Disease: Improving Global Outcomes
MAP: Mean arterial pressure
OHRPP: Office of the Human Research Protection Program
PAH: Pulmonary arterial hypertension
PH: Pulmonary hypertension
PVR: Pulmonary vascular resistance
RV: Right ventricle
SEP-1: Septic shock management bundle
SIRS: Systemic inflammatory response syndrome
SOFA: Sequential organ failure assessment
