# Supplementary material for: Management and outcomes in pulmonary arterial hypertension patients with sepsis
Source: BMC Pulm Med. 2024 Oct 28;24:538. doi: 10.1186/s12890-024-03355-5 (PMC11520816; doi:10.1186/s12890-024-03355-5)
Supplement: Supplementary file 1 — Supplementary Material 1 [file 12890_2024_3355_MOESM1_ESM.docx]

Supplemental File: Management and Outcomes in Pulmonary Arterial Hypertension Patients with Sepsis

ICD-10 codes to identify sepsis present on admission included A021, A227, A267, A327, A400, A401, A403, A408, A409, A4101, A4102, A411, A412, A413, A414, A4150, A4151, A4152, A4153, A4159, A4181, A4189, A419, A427, A5486, R6520, R6521

ICD-10 codes to identify a prior diagnosis of pulmonary hypertension included I27.0, I27.2, I27.20, I27.21, I27.22, I27.23, I27.24, I27.29, I27.8, I27.83, I27.89


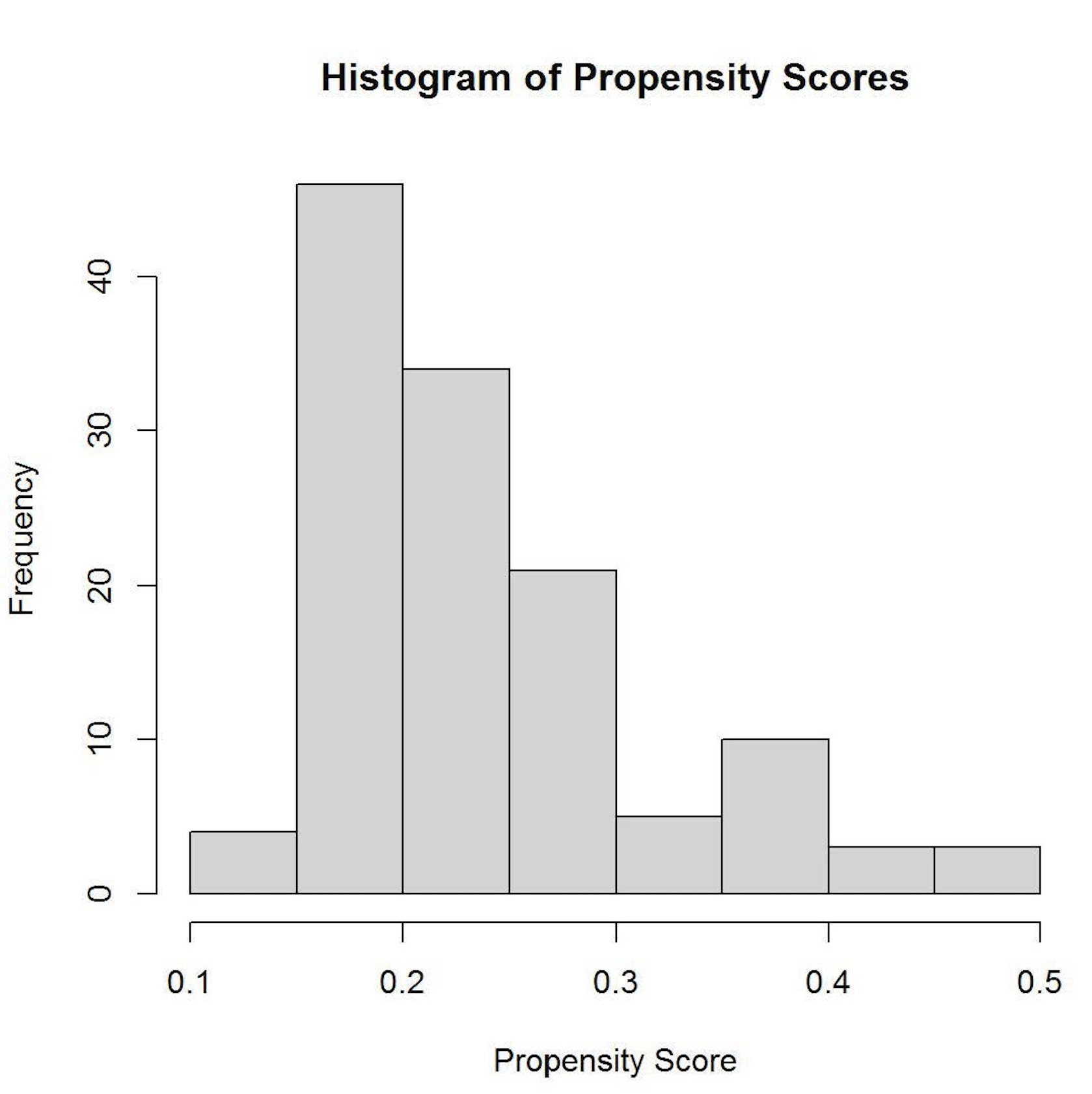


**Supplemental Figure 1:** Histogram of Propensity Scores

| Supplemental Table 1: Non-propensity Score Weighted Demographics and Clinical Characteristics of Patients with Pulmonary Arterial Hypertension Admitted for Sepsis Compared to Controls | | |  |
| --- | --- | --- | --- |
|  | Cases (n=30) | Controls (n=96) | P-value |
| Age, years | 58.5 ± 15.9 | 57.5 (16.4) | 0.759 |
| Sex, Female | 24 (80%) | 73 (76%) | 0.841 |
| Race  White  Black  Asian  Other | 15 (50%)  6 (20.0%)  1 (3.3%)  8 (26.6%) | 58 (60.4%)  5 (5.2%)  11 (11.5%)  22 (22.6%) | 0.399  **0.031**  **0.008**  0.818 |
| Charlson comorbidity index | 4.7 ± 3.0 | 3.7 ± 2.5 | 0.096 |
| Presenting vitals and laboratory data  MAP, mmHg  Respiratory rate, per minute  Temperature, Fahrenheit  Heart rate, per minute  SpO2, %  WBC, x10^3^/µL  Lactate, mg/dL  Creatinine, mg/dL  BNP, pg/mL | 77.1 ± 17.6  20.9 ± 5  98.7 ± 1.7  94.7 ± 21  92.2 ± 6.8  9.7 ± 5.6  16.3 ± 2.6  2.43 ± 2.64  859.2 ± 761.3 | 91.2 ± 16  20 ± 5.5  99.1 ± 2.3  101.1 ± 21.2  95.3 ± 7.4  14.3 ± 10.1  20.4 ± 19.5  1.46 ± 1.36  601 ± 779 | **<0.001**  0.410  0.440  0.153  **0.045**  **0.020**  0.288  **0.010**  0.180 |
| Blood culture, positive at 24 hours | 9 (30%) | 24 (25.2%) | 0.760 |
| Septic shock | 9 (30%) | 8 (8.0%) | **0.006** |
| PH medication during admission, categorical | 26 (86.7%) | N/A | N/A |
| Received parenteral PH medication during admission | 10 (33.3%) | N/A | N/A |
| Baseline PH medications used, categorical | 24 (80%) | N/A | N/A |
| Number of baseline PH medications used  0  1  2  3 | 6 (20%)  6 (20%)  5 (16.7%)  13 (43.4%) | N/A | N/A |
| Maximal supplemental oxygen use at 24 hours  ETT  PPV  HFNC  NRB  NC  Room air | 0  1 (3.3%)  10 (33.6%)  2 (6.6%)  13 (43.3%)  4 (13.3%) | 6 (6.3%)  4 (4.2%)  2 (2.1%)  8 (8.3%)  31 (32.3%)  45 (46.9%) | **0.012**  0.440  **<0.001**  0.594  0.276  **<0.001** |
| *Abbreviations:* MAP = mean arterial pressure, SpO2 = oxygen saturation, WBC = White blood cell count, BNP = brain natriuretic peptide, PH = pulmonary hypertension, ETT = endotracheal intubation, PPV = positive pressure ventilation, HFNC = high flow nasal cannula, NRB = non-rebreather mask, NC = nasal canula  All numeric values are reported as mean ± SD  All vital signs and lab values reported are the earliest available values during the qualifying encounter for sepsis. *p<0.05, **p<0.01, ***p<0.001 | | | |

| Supplemental Table 2: Etiology of Sepsis in Cases versus Controls | | | |
| --- | --- | --- | --- |
|  | PAH patients (n=30) | Controls (n=96) | P-value |
| Community acquired pneumonia | 7 (23.3%) | 20 (20.8%) | 0.771 |
| Hospital or ventilator acquired pneumonia | 1 (3.3%) | 7 (7.3%) | 0.438 |
| Upper respiratory infection | 5 (16.7%) | 6 (6.3%) | 0.078 |
| Gastrointestinal infection | 6 (20%) | 19 (19.8%) | 0.980 |
| Urinary tract infection | 3 (10%) | 17 (17.8%) | 0.313 |
| Endocarditis | 2 (6.7%) | 0 | **0.011** |
| Central line infection | 5 (16.7%) | 0 | **<0.001** |
| SSTI/osteomyelitis/dental | 1 (3.3%) | 11 (11.5%) | 0.186 |
| Bacteremia of unknown etiology | 0 | 5 (5.2%) | 0.202 |
| Other | 0 | 11 (11.5%) | 0.052 |
| *Abbreviations:* SSTI = Skin and soft tissue infections  *p<0.05, **p<0.01, ***p<0.001 | | | |

| Supplemental Table 3: Clinical Characteristics and Treatment Approaches of Patients with Pulmonary Arterial Hypertension Admitted for Sepsis by Baseline Right Ventricle Systolic Function | | | |
| --- | --- | --- | --- |
|  | RV systolic function normal (n=16) | RV systolic function reduced (n=14) | P-value |
| Age | 62.5 (54.5-74.5) | 53.5 (43-59) | 0.061 |
| Sex, female | 11 (68.8%) | 13 (92.9%) | 0.234 |
| Race  White  Black  Asian  Other | 8 (50%)  6 (37.5%)  0  2 (12.5%) | 7 (50%)  1 (7.1%)  1 (7.1%)  5 (35.7%) | 0.121 |
| Charlson comorbidity index | 5 (4.8-6.0) | 2.5 (2.0-6.2) | 0.208 |
| Presenting vitals and laboratory data  MAP, mmHg  Respiratory rate, per minute  Temperature, Fahrenheit  Heart rate, per minute  SpO2, %  WBC, x10^3^/µL  Serum lactate, mg/dL  BNP, pg/mL | 76 (69.3-84.6)  18 (16.8-20.0)  98.3 (98.0-99.4)  95 (76.5-104.2)  95 (92.8-96)  9 (5.6-13.9)  16 (9-20.0)  326.0 (139.0-673.0) | 69.3 (64.3-87.1)  20.0 (18.0-27.0)  98.0 (97.5-99.1)  103.5 (80.0-110.8)  93.5 (89.2-95.8)  9.2 (6-11.1)  10.5 (8.0-14.0)  961.5 (460.5-1399.2) | 0.677  0.091  0.546  0.394  0.380  0.967  0.053  **0.033** |
| Blood culture, positive | 5 (31.2%) | 4 (28.3%) | 0.274 |
| Sepsis category  Sepsis  Severe sepsis  Septic shock | 7 (43.8%)  6 (37.5%)  3 (18.8%) | 5 (35.7%)  3 (21.4%)  6 (42.9%) | 0.331 |
| PH medication during admission | 12 (75%) | 14 (100%) | 0.141 |
| Received parenteral PH medication during admission | 8 (50%) | 11 (78.6%) | 0.215 |
| Baseline PH medication use, yes | 11 (68.8%) | 14 (100%) | 0.072 |
| Number of baseline PH medications used  0  1  2  3 | 5 (31.2%)  2 (12.5%)  5 (31.2%)  4 (25%) | 0  5 (35.7%)  1 (7.1%)  8 (57.1%) | **0.017** |
| Supplemental oxygen use at 24 hours  None  NC  NRB  HFNC  PPV | 4 (25%)  7 (43.8%)  1 (6.2%)  4 (25%)  0 | 0  7 (50%)  1 (7.1%)  5 (35.7%)  1 (7.1%) | 0.220 |
| Time to antibiotics, hours | 2.9 (1.9-5.5) | 4.8 (2.1-8.1) | 0.850 |
| Most recent RHC findings,  PCWP, mmHg  Mean RAP, mmHg  Mean PAP, mmHG  PVR, WU  CI, thermodilution, L/min | 13.0 (9.0-18.0)  8.0 (5.0-10.2)  40.5 (32.5-46.0)  4.6 (3.2-7.0)  3.0 (2.6-3.2) | 10.5 (6.5-13.8)  7.5 (6.0-13.5)  45.0 (37.8-50.8)  8.4 (6.4-10.8)  2.4 (2.0-3.0) | 0.274  0.587  0.328  0.051  0.186 |
| TAPSE, cm | 1.9 (1.8-2.4) | 2.0 (1.7-2.2) | 0.930 |
| Change in lactate | -8.5 (-11.2, -6.0) | -3.0 (-6.0, -0.5) | 0.056 |
| Change in creatinine | 0.0 (-0.3, 0.1) | -0.1 (-0.2, 0.1) | 0.677 |
| AKI present on admission | 6 (37.5%) | 4 (28.6%) | 0.287 |
| DTI, cm/sec | 11.1 (10.8-15.0) | 11.7 (11.1-14.0) | 0.536 |
| Mechanical ventilation used during admission | 3 (18.8%) | 6 (42.9%) | 0.299 |
| IV fluid boluses in first 24 hours, mL | 250 (0-850) | 0 (0-374) | 0.114 |
| IV fluid boluses in first 48 hours, mL | 400 (0-1500) | 0 (0-575) | 0.192 |
| Received fluid bolus | 10 (62.5%) | 4 (28.6%) | 0.136 |
| Diuretic use in first 24 hours | 8 (50%) | 6 (42.9%) | 0.980 |
| Diuretic use in first 48 hours | 9 (56.2%) | 9 (64.3%) | 0.940 |
| Vasopressor use in first 24 hours | 3 (18.8%) | 4 (28.6%) | 0.840 |
| Vasopressor use in first 48 hours | 4 (25%) | 6 (42.9%) | 0.518 |
| Hospital length of stay | 8 (7-19) | 26 (12.5-64.8) | **0.011** |
| ICU stay during admission | 6 (37.5%) | 12 (85.7%) | **0.021** |
| ICU length of stay | 21.5 (13.5-26.5) | 26 (11.8-38.0) | 0.452 |
| *Abbreviations:* MAP = mean arterial pressure, SpO2 = oxygen saturation, WBC = White blood cell count, RAP = right atrial pressure, PAP = pulmonary artery pressure, PCWP = pulmonary capillary wedge pressure, CI = cardiac index, PVR = pulmonary vascular resistance, WU = Wood units  All numeric values are reported as median (IQR) and p-values are from Kruskal-Wallis tests.  All vital signs and lab values reported are the earliest available values during the qualifying encounter for sepsis. All quantitative variables are reported as median (IQR). *p<0.05, **p<0.01, ***p<0.001 | | | |

| Supplemental Table 4: Non-Propensity Score Weighted Treatment Approaches and Outcomes in Pulmonary Arterial Hypertension Patients Admitted for Sepsis Compared to Controls | | | |
| --- | --- | --- | --- |
|  | All PAH patients (n=30) | Controls(n=96) | P-value |
| IV fluid boluses in first 24 hours, mL, median (IQR) | 0 (0-750) | 1218 (0-3743.8) | **<0.001** |
| IV fluid boluses in first 48 hours, mL,  Median (IQR) | 250 (0-1412) | 1376 (0-4189) | **<0.001** |
| Vasopressor use in first 24 hours | 7 (23.3%) | 8 (8.3%) | **0.037** |
| Vasopressor use in first 48 hours | 10 (33.3%) | 8 (8.3%) | **0.004** |
| Hospital length of stay, days | 32.6 ± 49.8 | 17.3 ± 29.7 | 0.125 |
| Mechanical ventilation during admission | 9 (30%) | 22 (22.9%) | 0.433 |
| Mortality during admission or within 30 days | 7 (23.3%) | 12 (12.5%) | 0.248 |
| *Abbreviations:* IV = intravenous, PH = pulmonary hypertension  All numeric variables are mean ± SD unless otherwise indicated. | | | |

| Supplemental Table 5: Logistic Regression of Receiving Fluid Resuscitation Among Patients with Pulmonary Arterial Hypertension Hospitalized for | |
| --- | --- |
|  | OR (95% CI) |
| TAPSE, continuous | 2.21 (0.36-13.38), p=0.39 |
| TAPSE, < 17mm, n=5 | 0.61 (0.07-5.07), p=0.65 |
| RV systolic function at least mildly impaired, n=14 | **0.14 (0.02-0.99), p=0.05** |
| RV systolic function at least moderately impaired, n=9 | 0.19 (0.03-1.41), p=0.10 |
| DTI, continuous | 1.04 (0.65-1.66), p=0.87 |
| DTI < 12 cm/s, n=10 | 0.39 (0.04-4.02), p=0.43 |
| AKI initial | 2.30 (0.40-15.02), p=0.36 |
| Lactate initial | **1.23 (1.04-1.55), P = 0.049** |
| Change in lactate | 0.95 (0.75-1.08), p=0.53 |
| *Abbreviations:* TAPSE = tricuspid annular plane systolic excursion, RV = right ventricle, DTI =doppler tissue echocardiography  All values reported are odds ratios generated from logistic regression analyses adjusted for age, sex, and Charlson Comorbidity Index at admission. | |

| Supplemental Table 6: Clinical Characteristics and Treatment Approaches by Fluid Administration within 24 Hours | | | |
| --- | --- | --- | --- |
|  | No Fluids (n=16) | Received Fluids (n=14) | P-value |
| Age | 58 (52.8-67.2) | 57.5 (47-73.5) | 0.901 |
| Sex, female | 13 (81.2%) | 11 (78.6%) | 1.000 |
| Race  White  Black  Asian  Other | 8 (50%)  3 (18.8%)  0  5 (31.2%) | 7 (50%)  4 (28.6%)  1 (7.1%)  2 (14.3%) | 0.499 |
| Charlson comorbidity index | 4 (2-6) | 5 (2.2-6) | 0.659 |
| Presenting vitals and laboratory data  MAP, mmHg  Respiratory rate, per minute  Temperature, Fahrenheit  Heart rate, per minute  SpO2, %  WBC, x10^3^/µL  Serum lactate, mg/dL  BNP, pg/mL | 72 (66.3-85.6)  20 (17.5-22.5)  98 (97.7-98.6)  91.5 (78.5-104.2)  94 (88.5-95.2)  9.2 (5.7-11.1)  11.5 (9-14.2)  561 (347-1332.5) | 73.5 (66-84.7)  18.5 (18-23.8)  98.7 (98.1-101.2)  103.5 (83-110.8)  94.5 (93-96)  8.6 (6.2-14.1)  18 (8-23)  509 (216.5-1180.5) | 0.950  0.983  0.070  0.270  0.452  0.280  0.124  0.626 |
| Blood culture, positive | 6 (37.5%) | 3 (21.4%) | 0.144 |
| Septic shock | 5 (31.3%) | 4 (28.6%) | 0.873 |
| SEP-1 Bundle criteria | 8 (50%) | 6 (42.9%) | 0.980 |
| PH medication during admission | 15 (93.8%) | 11 (78.6%) | 0.495 |
| Received parenteral PH medication during admission | 14 (87.5%) | 5 (35.7%) | **0.011** |
| Baseline PH medication use, yes | 15 (93.8%) | 10 (71.4%) | 0.252 |
| Number of baseline PH medications used  0  1  2  3 | ­  1 (6.2%)  4 (25%)  3 (18.8%)  8 (50%) | 4 (28.6%)  3 (21.4%)  3 (21.4%)  4 (28.6%) | 0.368 |
| Supplemental oxygen use at 24 hours  None  NC  NRB  HFNC  PPV | 2 (12.5%)  5 (31.2%)  1 (6.2%)  7 (43.8%)  1 (6.2%) | 2 (14.3%)  9 (64.3%)  1 (7.1%)  2 (14.3%)  0 | 0.235 |
| Time to antibiotics, hours | 6.5 (2.5-19.1) | 2.3 (1.2-4.5) | **0.041** |
| Most recent RHC findings,  PCWP, mmHg  Mean RAP, mmHg  Mean PAP, mmHG  PVR, WU  CI, thermodilution, L/min | 13 (10.5-14.5)  8.5 (5-12.5)  45.5 (40-52)  7.9 (4.3-10.1)  2.8 (2.2-3.4) | 9.5 (7.2-13.8)  7.5 (5.2-10.8)  37 (27-45)  4.7 (3.4-8.2)  2.9 (2.3-3.1) | 0.265  0.602  **0.037**  0.270  0.982 |
| TAPSE, cm | 1.8 (1.8-2.0) | 2.2 (1.8-2.5) | 0.154 |
| RV systolic function  Normal  Mildly reduced  Moderately reduced  Severely reduced | 6 (37.5%)  3 (18.8%)  2 (12.5%)  5 (31.2%) | 9 (64.3%)  2 (14.2%)  1 (7.1%)  1 (7.1%) | 0.464 |
| RV systolic function reduced | 10 (62.5%) | 4 (28.6%) | 0.136 |
| Change in lactate | -5 (-6, -4) | -6.5 (-10.2, -0.8) | 0.613 |
| Change in creatinine at 24 hours | -0.1 (-0.2, 0) | 0.0 (-0.3, 0.1) | 0.539 |
| AKI present on admission | 4 (25%) | 6 (42.9%) | 0.560 |
| Resolution of AKI present on admission within 48 hours | 2 (13.3%) | 6 (42.9%) | 0.179 |
| DTI, cm/sec | 11.1 (11-14) | 12.9 (10.8-15.0) | 0.825 |
| Mechanical ventilation used during admission | 4 (25%) | 5 (35.7%) | 0.811 |
| IV fluid boluses in first 24 hours, mL | 0 (0-0) | 775 (512.5-1375.0) | **<0.001** |
| IV fluid boluses in first 48 hours, mL | 0 (0-0) | 1025 (512.5-1518.5) | **<0.001** |
| Diuretic use in first 24 hours | 9 (56.2%) | 5 (35.7%) | 0.448 |
| Diuretic use in first 48 hours | 13 (81.2%) | 5 (35.7%) | **0.030** |
| Vasopressor use in first 24 hours | 4 (25%) | 3 (21.4%) | 1.000 |
| Vasopressor use in first 48 hours | 7 (43.8%) | 3 (21.4%) | 0.365 |
| Hospital length of stay | 16 (11-36) | 9 (7-29.2) | 0.304 |
| ICU stay during admission | 11 (68.8%) | 7 (50%) | 0.501 |
| ICU length of stay | 22 (12-30) | 25 (13-33.5) | 1.000 |
| *Abbreviations:* MAP = mean arterial pressure, SpO2 = oxygen saturation, WBC = White blood cell count, RAP = right atrial pressure, PAP = pulmonary artery pressure, PCWP = pulmonary capillary wedge pressure, CI = cardiac index, PVR = pulmonary vascular resistance, WU = Wood units  All vital signs and lab values reported are the earliest available values during the qualifying encounter for sepsis.  All numeric values are reported as median (IQR) and p-values are from Kruskal-Wallis tests. *p<0.05, **p<0.01, ***p<0.001 | | | |
